# Four cases of cutaneous Rosai-Dorfman in Black patients: A review of a single institution’s experience with this rare disease

**DOI:** 10.1016/j.jdcr.2023.09.032

**Published:** 2023-10-11

**Authors:** Sara Banbury, Brian Chu, Matthew L. Hedberg, Temitayo A. Ogunleye, Ellen Kim, Misha Rosenbach

**Affiliations:** a Perelman School of Medicine at the University of Pennsylvania, Philadelphia, Pennsylvania; b Department of Medicine, California Pacific Medical Center, San Francisco, California; c Department of Dermatology, University of Pennsylvania, Philadelphia, Pennsylvania

**Keywords:** cutaneous Rosai-Dorfman, emperipolesis, histiocytosis, oct-2, Rosai-Dorfman disease

## Introduction

Rosai-Dorfman disease (RDD) is a rare non–Langerhans cell histiocytosis (non-LCH) in which an overabundance of activated histiocytes accumulate within tissues resulting in a clinical presentation that often includes massive bilateral cervical lymphadenopathy, fever, and leukocytosis.^1^ Cutaneous RDD (CRDD) is a skin-limited form of the disease with shared histological features but without systemic symptoms, lymphadenopathy, or other extranodal involvement which typically follows a benign clinical course.^2^ CRDD is even rarer than RDD, with approximately 200 cases reported in the literature.^3^

Cutaneous RDD (CRDD) presents with slowly progressive papules, nodules, plaques, and/or tumors, typically on the torso or head and neck region. Epidemiologically, it is distinct from systemic RDD with a mean age of presentation at 43.5 years, compared to 20.6, and more common in Whites and Asians, compared to Blacks, with an inverse male/female ratio (1.4:1 in systemic RDD and 1:1.8 in CRDD).^4^ Biopsy is required for definitive diagnosis of RDD. Histology shows enlarged, foamy histiocytes with emperipolesis. To diagnose RDD, these cells should express the histiocytic markers CD68 and CD163 as well as S100 and Oct-2 and must not express CD1a.^5^^,^^6^ Skin lesions can be the first manifestation of systemic disease, so close clinical follow-up is critical. Treatment options for CRDD include cryotherapy, excision, topical, intralesional or oral steroids, low dose methotrexate, and local radiation, but there is no consensus on optimal treatment and evidence remains limited.^2^ This paper presents 4 cases of CRDD at a single center, including diagnosis, management, and follow-up.

## Case descriptions

**Fig 1:**
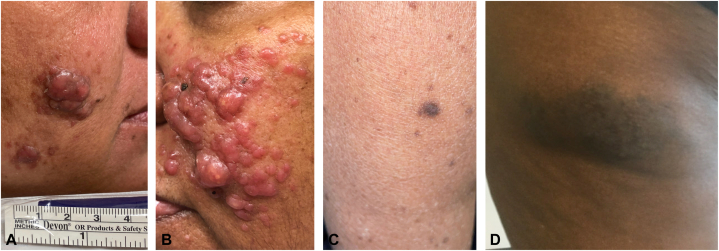
**A-D,** Cutaneous Rosai-Dorfman Disease (**A**) patient 1: *pink* nodules with surrounding papules and macules. **B,** Patient 2: *Pink* nodules and papules diffusely on face. **C,** Patient 3: *brown* papule on L thigh. **D,** Patient 4 irregular hyperpigmented nodule on L thigh.

**Fig 2:**
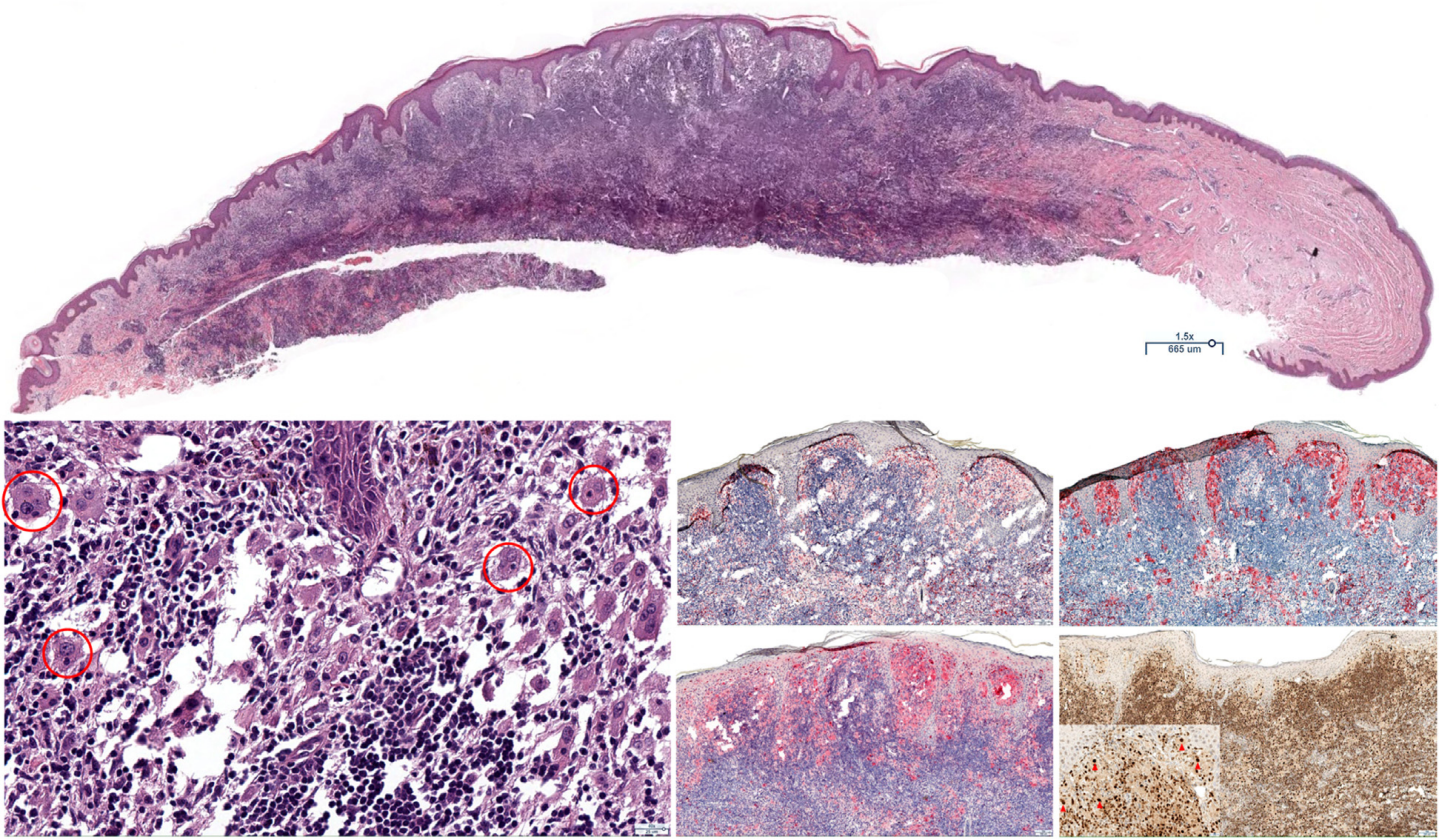
Patient 1, Biopsy 2, Abdomen: Staining with hematoxylin and eosin at scanning magnification (*top*) shows a dense and diffuse mixed infiltrate composed of lymphocytes and granulocytes with histiocytes scattered throughout the lesion as single cells and as aggregates superficially. Examination at high magnification (*lower left*) shows lymphocytes, neutrophils, and plasma cells amongst cytologically atypical histiocytes with abundant foamy cytoplasm and multiple nucleoli, a subset of which show emperipolesis (*circles*). The lesional histiocytes stain positively for CD68 (*middle center*), CD163 (*middle right*) and S100 (*bottom center*). Strong positivity is seen amongst the lesional histiocytes (*arrows*) and background B-cells for Oct-2 (*bottom right and inset*). The cells are negative for CD1a (not shown). These histologic and immunophenotypic characteristics are consistent with a diagnosis of Rosai-Dorfman Disease. Total original magnifications: 15× (*top left*), 100× (*middle center, middle right, bottom center, bottom right*) and 400× (*lower left and inset*).

Patient 1 is a Black woman in her late 50s with type 2 diabetes and hypertension who presented with 2 months of slowly growing small pink translucent papules on her face, abdomen, and knees (Fig 1, *A*) (Table I). She had no lymphadenopathy or systemic symptoms. She had 2 metachronous biopsies taken 1 year apart – both a punch and a shave biopsy (Fig 2). Both specimens showed a dense, diffuse, mixed infiltrate. Histocytes were positive for C68, CD163, and S100, though abundant cytoplasm depleted the intensity of the stains. Oct-2 positivity and the presence of emperipolesis confirmed RDD. Due to concerns for potential B-cell lymphoma, IgH rearrangement studies were performed and failed to demonstrate a clonal population. She was initially treated with intralesional steroids which did not lead to improvement. Her disease proved refractory, and she saw little to no improvement with multiple treatment modalities and has since been lost to follow up.

**Table I:** Rosai-Dorfman Disease case descriptions

Patient	Age	Race	Clinical presentation	Histology	Systemic workup	Genetic testing	Treatment approach and responses (−/+)
1	56	Black	Small pink translucent papules on face, abdomen, and knees	CD68+ CD163+ S100+ Oct2+ CD1a−	CT Chest/Neck/Abdomen/Pelvis with contrast negative. Infectious cultures CBC, thyroid studies, CMP, and ESR all unremarkable.	TCR gene rearrangement (−) IgH rearrangement (−) BRAF (−) MAPK (−) FGR1 (+)) Not performed	Intralesional steroids (−) Methotrexate 25 mg/week (−) Lenalidomide 15 mg with dexamethasone 40 mg (−) Rituximab (?)
2	73	Black	Pink and brown papules and nodules limited to face	S100+ CD1a−	CT Neck/Chest/Abdomen/Pelvis with contrast negative. Thyroid function labs, CBC, and CMP all unremarkable		Prednisone 5 mg daily (−) Betamethasone dipropionate 0.05 % cream (−) Tacrolimus 0.1% ointment (−) Methotrexate 10 mg/week (+) – stopped due to side effects Hydroxychloroquine 400 mg daily – stopped due to gastrointestinal upset Intralesional steroids (+)
3	75	Black	Pink brown nodules on her left upper thigh and hyperpigmented macules on bilateral lower extremities	S100+ CD68+ CD1a−	CT Neck/Chest/Abdomen/Pelvis with contrast negative. Thyroid function labs, CBC, CMP, LDH and QuantiFERON gold are all unremarkable	Not performed	Declined treatment
4	50	Black	Irregular firm hyperpigmented nodule on her lateral left thigh	S-100+ CD68 + (variable) CD1a-CD34−	CT Abd/Pelvis with contrast negative.∗ Chest x-ray negative. Serum protein electrophoresis within normal limits; elevated Kappa:Lambda ratio.		Minocycline 100 MG twice a day (−) Topical steroids (?) Surgical excision (+)

Key for Treatment responses: (−) no/insufficient response (+) significant improvement (?) lost to follow up or patient unable to recall.

∗ CT performed prior to confirmation of diagnosis, but 9 years after lesion appeared.

Patient 2 is a Black woman in her early 70s with type 2 diabetes, hypertension, and sleep apnea who presented with 10 months of slowly progressive pink and brown papules and nodules on her face (Fig 1, *B*) (Table I). She had no lymphadenopathy or systemic symptoms. A punch biopsy showed a dense infiltrate occupying the superficial to deep dermis, consisting of large cells with pale cytoplasm displaying emperipolesis, consistent with atypical histiocytes, lymphocytes, and plasma cells. The atypical histiocytes stained positively with S100 stains and negative for CD1a. The patient trialed multiple treatments before experiencing improvement with intralesional steroids.

Patient 3 is a Black woman in her mid 70s with a history of breast cancer, marginal zone lymphoma of mucosa-associated lymphoid tissue of stomach, cerebrovascular accident and type 2 diabetes who presented with multiple months of pink brown nodules on her left thigh and hyperpigmented macules on bilateral lower extremities (Fig 1, *C*) (Table I). She had no lymphadenopathy but did endorse fatigue and some cognitive slowing, both of which were attributed to her previous stroke. A shave biopsy showed a dense infiltrate in the dermis characterized by large epithelioid cells with an abundance of cytoplasm and small vesicular nuclei showing emperipolesis. An admixture of neutrophils, lymphocytes, and plasma cells were seen. The large histiocytoid cells showed weak to moderate positivity for CD68 and strong positivity for S100, and were negative for CD1a. The lesions were asymptomatic, and she chose not to pursue treatment.

Patient 4 is a Black woman in her early 50s with a history of type 2 diabetes, hypertension, and obesity who presented with 11 years of an irregular hyperpigmented nodule on her lateral left thigh (Fig 1, *D*) (Table I). She had no lymphadenopathy or systemic symptoms. An excisional biopsy showed a dense dermal mixed inflammatory reaction pattern composed of lymphocytes, significant numbers of plasma cells, histiocytes, and multinucleated giant cells. Multiple areas of emperipolesis are present with a fibrotic and spindled cell component throughout the process. The areas of emperipolesis highlight with S-100 and variable CD68. CD1a negative within these histiocytes and CD34 is negative within the spindle cell regions. There was an elevated Kappa:Lambda ratio. Initial superficial outside biopsy was read as granulomatous inflammation, and treatment was attempted with topical steroids. On presentation to our clinic minocycline was trialed with symptomatic improvement but no visible change in the lesion. Diagnostic excisional biopsy was curative.

## Discussion

Here, we present 4 cases of cutaneous RDD at a single center. Each patient had variable response to treatment, demonstrating the clinical heterogeneity within this rare disease. The association between RDD and lymphoma has previously been described, and one patient in this series had her lymphoma at a distinct site and was in remission at the time of diagnosis.^5^ All 4 of our patients were Black, which is atypical for cutaneous RDD. These patients represent all the cases of cutaneous RDD identified at our institution through systemic evaluative search of a pathology database and interviews with providers. These cases provide valuable clinical images of CRDD in skin of color. A thorough clinical and radiologic evaluation is necessary in any patient presenting with skin lesions consistent with RDD, regardless of rate or ethnicity.

The value of Oct-2 as a helpful diagnostic marker in Rosai-Dorfman is also highlighted by our study in cases where the atypical histiocytes may have weak reactivity to histiocytic lineage markers.

Additionally, the 2 patients who pursued treatment had disease that proved refractory to numerous therapies, and one patient did escalate to a biologic agent with rituximab infusions. The sample size is small, but given the rarity of the disease, it provides important evidence of the difficulty treating this disease.

There have been recent advancements in our understanding of RDD and the molecular pathways that are impacted, such as the MAPK pathway.^5^ In other histiocytosis, similar discoveries have led to targeted therapy approaches.^7^ In one study, patients with RDD and Erdheim-Chester disease (ECD), a different non-LCH, MEK inhibitor trametinib had a 71% response rate in patients both with and without the BRAFV600E mutation.^8^ While the mutation analysis in Case 1 was negative for MAPK mutations, and did not lead to a change in treatment, more advanced understanding of RDD may lead to future more targeted and/or more effective therapeutic options.

## Conflicts of interest

None disclosed.
